# Supplementary material for: Functional connectivity changes in the brain of adolescents with internet addiction: A systematic literature review of imaging studies
Source: PLOS Ment Health. 2024 Jun 4;1(1):e0000022. doi: 10.1371/journal.pmen.0000022 (PMC12798305; doi:10.1371/journal.pmen.0000022)
Supplement: S1 Appendix — (DOCX) [file pmen.0000022.s002.docx]

**Supplementary Materials**

**Functional connectivity changes in the brain of adolescents with internet addiction: A systematic literature review of imaging studies**

**Search Terminology**

The databases mentioned in the article were searched using a range of terms. The search terms chosen had a direct relationship to the study title and objectives and were derived from earlier literature on the subject. Advanced searching was performed in the databases, and many search words were entered at once.

Terms for internet addiction:

Internet addiction, internet dependence, pathological internet use, online gaming addiction, compulsive computer use.

Terms for neurological components:

Dopaminergic, rewards pathway, rewards processing, mesolimbic, mesocortical, executive functioning, triadic model of development, loss avoidance, rewards seeking, impulsivity, compulsivity, functional connectivity, functional connectivity and executive control/ rewards pathway, genetics, glutamate, Gaba pathway, rewards reliance, mri, resting state, neurotransmitters, children, and prefrontal cortex.

Terms for adolescent:

Adolescents, adolescent behaviour, adolescent development, adolescent psychology.
