# Supplementary material for: Functional connectivity changes in the brain of adolescents with internet addiction: A systematic literature review of imaging studies
Source: PLOS Ment Health. 2024 Jun 4;1(1):e0000022. doi: 10.1371/journal.pmen.0000022 (PMC12798305; doi:10.1371/journal.pmen.0000022)
Supplement: Attachment — Submitted filename: PLOS MH_Authors response to Reviewers.docx [file pmen.0000022.s004.docx]

**The authors’ responses to the reviewers and editor’s comments and suggestions** (in purple) for the manuscript **PMEN-D-23-00073** titled “Functional connectivity changes in the brain of adolescents with internet addiction: A systematic literature review of imaging studies”

**Journal Requirements:**

1. Please note that your Data Availability Statement is currently missing [the repository name and/or the DOI/accession number of each dataset OR a direct link to access each database]. If your manuscript is accepted for publication, you will be asked to provide these details on a very short timeline. We therefore suggest that you provide this information now, though we will not hold up the peer review process if you are unable.

Thank you for this suggestion in advance. We have added Data Availability Statement in the Title Page and provided a supplementary file with all the searched papers from the databases PubMed and PsycINFO as well as the final reviewed 12 articles. The file has contained all the direct links to access each database and the categorised content of the articles for the screening process.

2. We have noticed that you have uploaded Supporting Information files, but you have not included a list of legends. Please add a full list of legends for your Supporting Information files after the references list.

We have now added the legends of the Supporting Information files after the reference list.

Additional Editor Comments (if provided):

[Note: HTML markup is below. Please do not edit.]

Reviewers' comments:

Reviewer's Responses to Questions

**Comments to the Author**
1. Does this manuscript meet PLOS Mental Health’s [publication criteria](https://eur01.safelinks.protection.outlook.com/?url=https%3A%2F%2Fjournals.plos.org%2Fmentalhealth%2Fs%2Fcriteria-for-publication&data=05%7C02%7Cirene.lee%40ucl.ac.uk%7C2503df2f241b4c7da2d308dc271b38f7%7C1faf88fea9984c5b93c9210a11d9a5c2%7C0%7C0%7C638428245069972802%7CUnknown%7CTWFpbGZsb3d8eyJWIjoiMC4wLjAwMDAiLCJQIjoiV2luMzIiLCJBTiI6Ik1haWwiLCJXVCI6Mn0%3D%7C0%7C%7C%7C&sdata=dFjd5V4QTJkzEoLuVrJIcpMvay0GATJsdvFbE9I6pg0%3D&reserved=0)? Is the manuscript technically sound, and do the data support the conclusions? The manuscript must describe methodologically and ethically rigorous research with conclusions that are appropriately drawn based on the data presented.

Reviewer #1: Yes

Reviewer #2: Yes

Reviewer #3: Yes

No responses from the authors are needed.

2. Has the statistical analysis been performed appropriately and rigorously?

Reviewer #1: No

Meta-analysis, as suggested by the reviewer #1, is not suitable for this review owing to the very low numbers of articles (1 or 2 articles, see Table 2) reporting the alterations of functional connectivity at different brain locations in participants with internet addiction. Therefore, statistical analysis was not applicable to this systematic review.

Reviewer #2: N/A

Reviewer #3: N/A

3. Have the authors made all data underlying the findings in their manuscript fully available (please refer to the Data Availability Statement at the start of the manuscript PDF file)?

The [PLOS Data policy](https://eur01.safelinks.protection.outlook.com/?url=https%3A%2F%2Fjournals.plos.org%2Fmentalhealth%2Fs%2Fdata-availability&data=05%7C02%7Cirene.lee%40ucl.ac.uk%7C2503df2f241b4c7da2d308dc271b38f7%7C1faf88fea9984c5b93c9210a11d9a5c2%7C0%7C0%7C638428245069984031%7CUnknown%7CTWFpbGZsb3d8eyJWIjoiMC4wLjAwMDAiLCJQIjoiV2luMzIiLCJBTiI6Ik1haWwiLCJXVCI6Mn0%3D%7C0%7C%7C%7C&sdata=sReH7bA8S94zKY0fdAKTgdbyJk9m7i76YfJy4VUcGxs%3D&reserved=0) requires authors to make all data underlying the findings described in their manuscript fully available without restriction, with rare exception. The data should be provided as part of the manuscript or its supporting information or deposited to a public repository. For example, in addition to summary statistics, the data points behind means, medians and variance measures should be available. If there are restrictions on publicly sharing data—e.g. participant privacy or use of data from a third party—those must be specified.

Reviewer #1: No

Reviewer #2: Yes

Reviewer #3: No

We have provided all the searched articles in the supplementary file S3 Data Records with all the details of categorised content for the screening process.

4. Is the manuscript presented in an intelligible fashion and written in standard English?

PLOS Mental Health does not copyedit accepted manuscripts, so the language in submitted articles must be clear, correct, and unambiguous. Any typographical or grammatical errors should be corrected at revision, so please note any specific errors here.

Reviewer #1: Yes

Reviewer #2: Yes

Reviewer #3: Yes

No responses from the authors are needed.

5. Review Comments to the Author

Please use the space provided to explain your answers to the questions above. You may also include additional comments for the author, including concerns about dual publication, research ethics, or publication ethics. (Please upload your review as an attachment if it exceeds 20,000 characters)

**Reviewer #1:** The manuscript addresses a contemporary and significant issue, namely internet addiction among adolescents. The rising global internet usage and its impact on neural networks make this study relevant and timely. The systematic search from reputable databases (PubMed and PsycINFO) and the strict adherence to inclusion/exclusion criteria, particularly regarding the adolescent age range and formal diagnosis of internet addiction, contribute to the methodological rigor of the review. The literature review covers both resting-state and task-based functional magnetic resonance imaging (fMRI) studies, providing a comprehensive overview of the consequences of internet addiction on the functional connectivity in the adolescent brain. The fMRI results are succinctly presented, detailing the effects of internet addiction on various neural networks. The delineation of increases/decreases in functional connectivity within specific networks adds clarity to the findings. The manuscript effectively connects changes in functional connectivity to subsequent behavioral alterations in adolescents, emphasizing the relevance of the neural changes to addictive behavior and tendencies.

Thank you, Reviewer #1, for the insightful comments which will improve the quality of the article.

However there are certain areas that need to be improved in the manuscript are listed below:

1. The discussion could be enhanced by delving deeper into the implications of the findings. Elaborating on how the observed changes in functional connectivity may influence specific behaviors and developmental aspects would provide a more nuanced understanding.

The discussion regarding the behavioural and developmental impact of the functional connectivity alterations has been strengthened by adding the results of another two studies relating to this topic. One study connected functional connectivity with attachment styles in adolescents, an important topic that influences adolescent behaviour and development. The other study depicted resting state functional connectivity differences in adolescents belonging to normal and disadvantaged neighbourhoods which further correlated with various levels of mental health. These observations emphasised how functional connectivity alterations are an important lens to view various neurological disorders. Through these additions, the readers will have a fuller understanding of the findings and their implications.

This has been added in the Discussion:

“*The implications of these findings towards adolescent behaviour are significant. Although the exact changes and mechanisms are not fully clear, the observed changes in functional connectivity have the capacity of influencing several aspects of adolescent development. For example, functional connectivity has been utilised to investigate attachment styles in adolescents (60). It was observed that adolescent attachment styles were negatively associated with caudate-prefrontal connectivity, but positively with the putamen-visual area connectivity (60). Both named areas were also influenced by the onset of internet addiction, possibly providing a connection between the two. Another study associated neighbourhood/socioeconomic disadvantage with functional connectivity alterations in the DMN and dorsal attention network (61). The study also found multivariate brain behaviour relationships between the altered/disadvantaged functional connectivity and mental health and cognition (61). This conclusion supports the notion that the functional connectivity alterations observed in IA are associated with specific adolescent behaviours as well as the fact that functional connectivity can be utilised as a platform onto which to compare various neurologic conditions.*”

2. It would be beneficial to explicitly acknowledge any limitations of the reviewed studies and the broader implications for the interpretation of the results. Discussing potential biases or methodological constraints would strengthen the manuscript.

We have listed several limitations, but after a review of the limitations section of the 12 included studies, small sample size and the inherent nature of cross-sectional studies were narrowed down as the most frequent in the articles. As a result, a section regarding those two limitations as well as their implications was added for further clarification.

This has been added and edited in the Limitation/Strength:

“*Reviewing the explicit limitations in each of the included studies, two major limitations were brought up in many of the articles. One was relating to the cross-sectional nature of the included studies. Due to the inherent qualities of a cross-sectional study, the studies did not provide clear evidence that IA played a causal role towards the development of the adolescent brain. While several biopsychosocial factors mediate these interactions, task-based measures that combine executive functions with imaging results reinforce the assumed connection between the two that is utilised by the papers studying IA. Another limitation regarded the small sample size of the included studies, which averaged to around 20 participants. The small sample size can influence the generalisation of the results as well as the effectiveness of statistical analyses. Ultimately, both included study specific limitations illustrate the need for future studies to clarify the causal relationship between the alterations of FC and the development of IA.*”

3. Including a section on potential future research directions or interventions based on the findings would add value to the manuscript. This could stimulate further discourse on addressing internet addiction in adolescents.

The manuscript already has a section regarding future directions/interventions. It was pointed out the need for more task-based resting state functional connectivity studies, comorbid studies, and the implications of bupropion treatment on adolescents. In the limitations section above it, the necessity of both larger sample-size studies as well as studies outside Far East Asia was included.

This has been added for the future research directions:

“*Future research on IA will aim to better understand the causal relationship between IA and psychological symptoms that coincide with it. The current literature regarding functional connectivity changes in adolescents is limited and requires future studies to test with larger sample sizes, comorbid populations, and populations outside Far East Asia.*”

4. The conclusion could be strengthened by summarizing the key insights and emphasizing the broader implications for future research, clinical applications, or public health interventions.

Several additional points have been added to the Conclusion. Firstly, a summary of what the future direction of IA research would look like, along with the specific limitations that are seen in today’s literature. Secondly, another section on the implications of future IA research on clinical practice and treatment was added, along with a potential public health intervention.

This has been added for the Conclusions:

And *“A clearer depiction of the mechanisms of IA would allow physicians to screen and treat the onset of IA more effectively. Clinically, this could be in the form of more streamlined and accurate sessions of CBT or family therapy, targeting key symptoms of IA. Alternatively clinicians could potentially prescribe treatment such as bupropion to target FC in certain regions of the brain. Furthermore, parental education on IA is another possible avenue of prevention from a public health standpoint. Parents who are aware of the early signs and onset of IA will more effectively handle screen time, impulsivity, and minimize the risk factors surrounding IA.”*

**Reviewer #2**: The manuscript was well written and presented in an intelligible manner. Very interesting topic and I believed that the article will contribute more to the field of internet addiction vs adolescent mental health. I think the manuscript should be published without any revision.

Thank you, Reviewer #2, for the positive comments which are very encouraging.

**Reviewer #3**: Thank you for submitting your work to Plos Mental health journal.
The research challenge is not adequately addressed in the introduction section. rather than providing a basic summary, researchers would be better served by doing a meta-analysis to address any issues that were either overlooked or not addressed in the original studies. A comprehensive meta-analysis would allow researchers to examine the functional connectivity changes in the brains of adolescents with internet addiction, while also providing an in-depth analysis of the pros and cons of related issues. This approach would provide a more thorough understanding of the topic and help to identify any gaps or limitations in the existing literature. By conducting a comprehensive meta-analysis, researchers can not only delve into the functional connectivity changes in the brains of adolescents with internet addiction but also explore a wide range of related topics, such as the impact of internet addiction on cognitive function, mental health, and social interactions. This approach would offer a more comprehensive understanding of the subject matter and enable the identification of any gaps or limitations in the existing literature, thus contributing to the advancement of research in this field.

Thank you, Reviewer #3, for the valuable comments and suggestions, which have been considered and checked if there were enough articles measuring at the same brain regions for conducting a meta-analysis.

However, during the screening process of this review, it was noted that there were several components to the search that limited the number of studies included. The main factor was the age restriction. Many IA related studies utilise the university age or young adult threshold. However, this review was primarily an adolescent study, so cutting the age limit at 19 excluded many studies. Furthermore, the frequency of literature on functional connectivity changes due to IA hasn’t been spread on a global scale and is limited only to Far East Asia. Due to these limiting factors, the review only included 12 studies that met the criteria. The results measuring at the same brain regions were reported in either 1 or 2 articles (see Table 2 in the manuscript). As a result, there was not enough articles to provide ample data for a meta-analysis.

In the “Limitations/Strengths” section, we have mentioned this as a limitation, “*Firstly, the study followed a systematic literature review design when analysing the fMRI studies. The data pulled from these imaging studies were namely qualitative and were subject to bias contrasting the quantitative nature of statistical analysis. Components of the study, such as sample sizes, effect sizes, and demographics were not weighted or controlled.*”

The study analyzed functional connectivity changes in the brains of adolescents with internet addiction, but no thematic analysis was presented. Without presenting any thematic analysis, it was difficult to draw conclusions from the study. An in-depth thematic analysis would have allowed the researchers to identify patterns and trends in the functional connectivity changes, which would have allowed them to better understand the causes and effects of internet addiction. Further research is needed to explore the specific neural networks affected by internet addiction and their implications for cognitive and emotional functioning. This is because the thematic analysis would have allowed the researchers to extract the key themes and patterns from the data, which could then be interpreted and used to draw meaningful conclusions. Additionally, an in-depth analysis of the neural networks affected by internet addiction could provide a better understanding of how internet addiction affects cognitive and emotional functioning. Furthermore, global reviews and meta-analyses could be included to derive distinct viewpoints and consolidate findings across multiple studies. This would enhance the robustness of the conclusions and provide a more comprehensive understanding of the functional connectivity changes associated with internet addiction in adolescents.

From the 12 reviewed articles, we have extracted all the informative data mentioning about the alterations of functional connectivity (FC) by internet addiction at various brain regions, listed the functions of those brain areas and presented the effects, including behavioural and developmental impact from the changes of FC at those regions (in the Results section for answering Research Questions 1 and 2 respectively). These findings have all been summarised in Table 2 and elaborated in the Results and Discussion. This was very similar to a thematic analysis and hence, we draw conclusions from these findings, from which we have a comprehensive understanding of FC alterations in adolescents with internet addiction.
